# The effect of a pressure ventilatory support on quadriceps endurance is maintained after exercise training in severe COPD patients. A longitudinal randomized, cross over study

**DOI:** 10.3389/fphys.2022.1055023

**Published:** 2022-11-28

**Authors:** Pierre Labeix, Isabelle Court Fortune, Daniela Muti, Mathieu Berger, Stéphanie Chomette-Ballereau, Jean Claude Barthelemy, Léonard Féasson, Frédéric Costes

**Affiliations:** ^1^ Department of Clinical and Exercise Physiology, University Hospital Center, Saint-Etienne, France; ^2^ INSERM, U1059, SAINBIOSE, DVH, Lyon University, Jean Monnet University, Saint-Etienne, France; ^3^ Pneumonology and Thoracic Oncology Department, University Hospital of Saint-Etienne, Saint-Etienne, France; ^4^ Cardiopulmonology Clinic of Durtol, Durtol, France; ^5^ Interuniversity Laboratory of Human Movement Biology, Lyon University, Jean Monnet University, Saint-Etienne, France; ^6^ Clermont Auvergne University, INRAE, Human Nutrition Unit, Clermont-Ferrand, France; ^7^ Department of Sports Medicine and Functional Explorations, University Hospital of Clermont-Ferrand, Clermont-Ferrand, France

**Keywords:** limb muscle, non-invasive ventilation, fatigue, pulmonary rehabilitation, COPD-chronic obstructive pulmonary disease

## Abstract

**Purpose:** In severe chronic obstructive pulmonary disease (COPD) patients, the application of an inspiratory pressure support (IPS) during exercise increases exercise tolerance and the benefit of exercise training during pulmonary rehabilitation (PR). Moreover, it improves quadriceps endurance after a session of cycling exercise suggesting a reduced muscle fatigue. We looked for the persistence of this effect after PR and sought an association between the improved quadriceps endurance with IPS and the training load during PR.

**Patients and methods:** We studied 20 patients with severe COPD (6 in stage 3and 14 in stage 4 of GOLD) before and after PR. As part of a PR program, patients completed 16 cycling sessions over 6 weeks with the addition of IPS during exercise. As a surrogate of muscular fatigue, quadriceps endurance was measured at 70% of maximal strength in a control condition, after a constant work rate exercise test (CWR) with IPS (TlimQ IPS) or with a sham ventilation (TlimQsham), in a random order. These tests were repeated similarly at the end of PR.

**Results:** PR was associated with a significant increase in maximal power output, cycling endurance, quadriceps strength and endurance. Session training load (power output x duration of the session) increased by 142% during the course of the program. Before PR, CWR duration increases with IPS compared to sham ventilation (Δtime = +244s, *p* = 0.001). Compared to control condition, post-exercise TlimQ reduction was lower with IPS at isotime than at the end of CWR or than with sham ventilation (−9 ± 21%, −18 ± 16% and −23 ± 18%, respectively, *p* = 0.09, *p* < 0.0001 and *p* < 0.0001). After PR, the post-exercise decrease of TlimQ was reduced after IPS compared to sham (−9 ± 18% vs. −21 ± 17%, respectively, *p* = 0.004). No relationship was found between the prevention of quadriceps fatigue and the training load.

**Conclusion:** In severe COPD patients, the beneficial effect of a ventilator support on quadriceps endurance persisted after PR with IPS. However, it was not related to the increase in training load, and could not predict the training response to non-invasive ventilation during exercise.

## Introduction

Pulmonary rehabilitation (PR) is a validated treatment which improves quality of life, increases exercise capacity and decreases dyspnea in patients with Chronic Obstructive Pulmonary Disease (COPD) whatever the severity of the disease (McCarthy et al., 2015). In the most disabled patients, non-invasive ventilation (NIV) as an add-on to exercise training proved to further increase maximal exercise capacity compared to usual exercise training with air breathing (Menadue et al., 2014).

When applied during exercise, NIV decreases dynamic hyperinflation which in turn decreases dyspnea and improves exercise tolerance (Hussain et al., 2011; Gagnon et al., 2014). Moreover, the reduction of the work of breathing decreases peripheral muscle fatigue in healthy subjects as well as in COPD patients (Amann et al., 2010; Bachasson et al., 2013).To explain the interplay between respiratory and limb muscles during exercise, Dempsey (Dempsey et al., 2006) proposed the existence of a metaboreflex which limits O2 delivery to peripheral muscles when the diaphragm is over burdened. In line with this, in COPD patients, applying NIV during exercise improved quadriceps O2 delivery as suggested by a decrease in the muscle sympathetic nerve activity (Haarmann et al., 2017) or a higher muscle oxygenation (Borghi-Silva et al., 2008). However, in a recent study, Goeckl et al. (Gloeckl et al., 2019) did not confirm this result during cycling with high level NIV compared to air breathing although exercise tolerance was improved. This suggests that the effect of NIV on exercise tolerance is multifactorial.

To further examine the effect of unloading the respiratory muscles on the exercise-induced variation in quadriceps function, we previously showed that quadriceps endurance, as a surrogate of post-exercise quadriceps fatigue, was higher when the exercise test was performed with an inspiratory pressure support (IPS) compared to ambient air breathing (Labeix et al., 2019). Since muscle deconditioning is a common feature in COPD patients, it could interfere with the response to respiratory muscle unloading. Exercise training, as a part of Pulmonary Rehabilitation program increases quadriceps endurance and lowers post-exercise fatigue (Mador et al., 2001; Marillier et al., 2019). So, we reasoned that the effect of IPS to reduce post-exercise quadriceps fatigue would be blunted after an exercise training program with NIV due to a less pronounced effect of the metaboreflex originating in the diaphragm.

Hence, we designed a crossover study; 1) to confirm previous results in a randomized order of the tests with the patients blinded of the experimental condition; 2) to assess whether the preventive effect of IPS on quadriceps fatigue was still present after exercise training; 3) to seek for a relationship between the NIV-induced prevention of post-exercise quadriceps fatigue and the training load during the PR program with the adjunct of a non-invasive ventilation.

## Material and methods

### Ethics approval and consent to participants

The study was performed from July 2015 to January 2019. Approval and ethical clearance was obtained from our local ethics committee (CPP Sud Est I 2014-A00145-42) which was in accordance with the principles embodied in the Declaration of Helsinki. Prior to study initiation, the objectives and purpose of the study were clearly explained to subjects in order to obtain written informed consent. The study was registered on ClinicalTrial (NCT02506504).

### Patients selection

**FIGURE 1:**
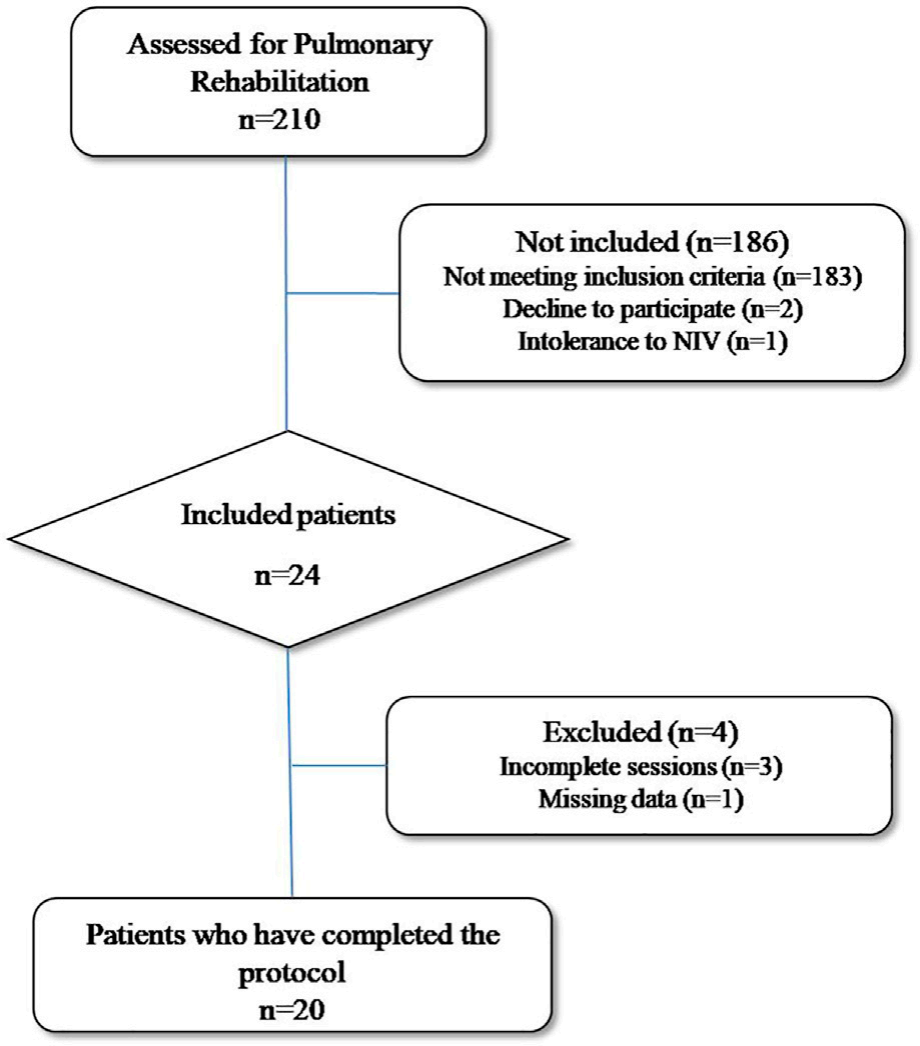
Flowchart of the study (NIV: Non Invasive Ventilation).

Amongst COPD patients referred to our center for a pulmonary rehabilitation program, we recruited those with a poor exercise capacity who could benefit of NIV during exercise training. Since no clear criteria for NIV adjunct emerged in the literature, we arbitrarily include patients presenting either a ventilatory limitation to exercise (see criteria below) or an inability to achieve a maximal power output above 50 W during the initial cardio-pulmonary exercise test (CPET). This cut-off of 50 W has been chosen according to our previous work (mean power output 46 W) (Labeix et al., 2019) and the averaged maximal power output ranging from 42 to 56 W reported in similar studies (Hawkins et al., 2002; Costes et al., 2003; van ’t Hul et al., 2006; Gloeckl et al., 2019). All were in a stable condition. The flow chart of the study is given in Figure 1.

### Functional evaluations

At inclusion in the program, we measured pulmonary function tests (BodyboxMedisoft, Sorinnes, Belgium) and resting arterial blood gases (ABL825, Radiometer, Copenhagen, Denmark). Body composition (fat free mass) was measured by bio-impedancemetry (Nutrigard,Germany) in standardized conditions and fat free mass was calculated according to Kyle’s equation (Kyle et al., 2001). Maximal power output (Wmax) and peak oxygen consumption (peak VO_2_) were determined during an incremental CPET on a cycloergometer with breath to breath gas exchange analysis (Ergocard, Medisoft, Sorinnes, Belgium). A ventilatory limitation to exercise was defined as a ventilatory reserve [(VEmaxpred–VEmax measured)/VEmaxpred] < 20% and/or as an exercise-induced CO_2_ retention >5 mmHg (American Thoracic Society and American College of Chest Physicians, 2003). On a separate day, a 6-min walk test was performed in duplicate according to recommendations and the longer distance was recorded (Singh et al., 2014).

Maximal voluntary quadriceps strength was performed as previously described (Labeix et al., 2019). Briefly, we measured the quadriceps maximal isometric force of the dominant thigh, in a 90° knee flexion position. Peak force was determined by a fixed strain gauge attached above the ankle (Globus, Codogné, Italy); three reproducible measurements (±5%) were performed and we recorded the highest value. Then, we determined one maximal repetition (1RM) by progressively increasing the load (van ’t Hul et al., 2004; Kraemer, WJ et al., 2006).

On separate days, the patients, blinded of the condition, performed constant workrate cycling tests (CWR) at 75% of Wmax until exhaustion with a pressure support or with a sham ventilation, in a random order. Pulse oximetry, transcutaneous carbon dioxide partial pressure (PtcCO_2_) and heart rate (Sentec, Basel, Switzerland) were monitored continuously during the test. Symptoms (dyspnea and fatigue) were evaluated at rest, after warm-up and at exhaustion, using a 10 points modified Borg scale.

### Non-invasive ventilation

NIV was set up during a preliminary habituation test, through a nasal mask (Elisée 150, ResMed, Saime, France). At rest, positive end expiratory pressure (PEEP) beginning at 4 cmH_2_O was gradually increased up to the maximum value tolerated by the patient. We first set inspiratory pressure support (IPS) to 10 cmH_2_O and thereafter, IPS was adjusted during CWR exercise in order to maintain dyspnea score below 5 on the10-points Borg scale. During the cycling exercise we also adjusted the ramp and the expiratory trigger according to the patients’ sensations (comfort to breathe and inspiratory time). When necessary, supplemental oxygen was added in order to maintain SpO2 ≥ 90%; in this case, the same oxygen flow rate was applied during all the tests and the training sessions. The set up of NIV was supervised by the same physiotherapist (PL).

Sham ventilation settings were the same for all the patients, PEEP was set to 2 cmH_2_O and IPS to 5 cmH_2_O according to Van’t Hul’s study (van ’t Hul et al., 2004). Pressure support lower than 8 cmH_2_O could be considered insufficient to alleviate the work of breathing for COPD patients (Brochard et al., 1991).

### Quadriceps endurance tests

A quadriceps endurance test was performed in a standardized position with 90° knee and hip flexions, and the arms crossed over the chest. The test was adapted from Serres et al. (Serres et al., 1998) and consisted in extending the dominant leg against a weight corresponding to 70% of 1RM with a pace of 12 movements per minute until exhaustion (duty cycle 40%). The test was stopped when the subject could no longer follow the movement amplitude or frequency on two consecutive movements despite strong verbal encouragements. The duration was then recorded as the quadriceps endurance limit time (TlimQ, seconds).

**FIGURE 2:**
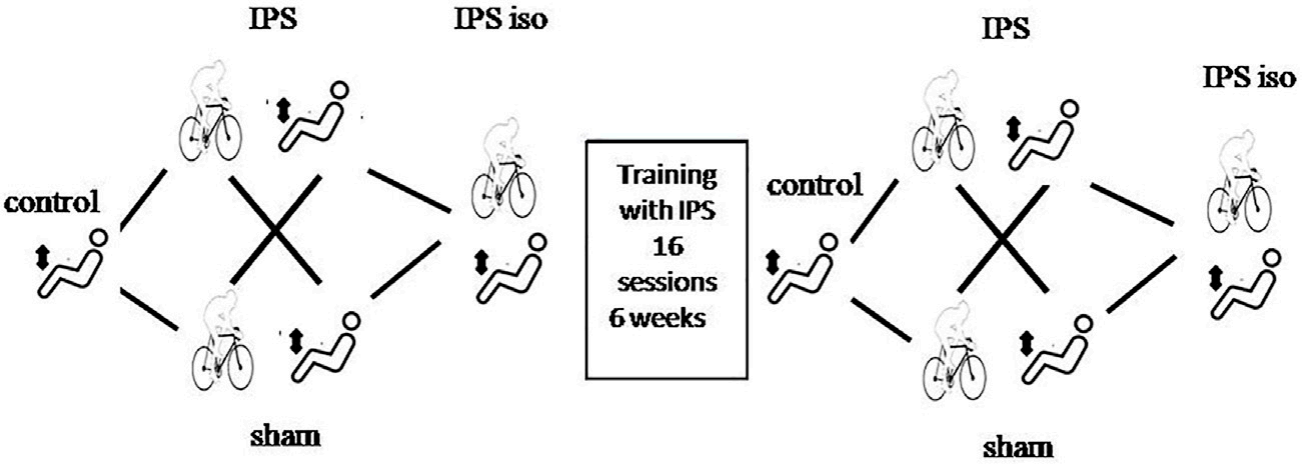
Study design. Quadriceps endurance (TlimQ) was assessed without (control) and after a constant workrate cycling exercise with effective ventilation (IPS) or sham ventilation in a random order. The tests were repeated similarly after completion of the pulmonary rehabilitation program.

In order to evaluate the effect on quadriceps endurance of a prior cycling exercise with or without NIV, we designed the following series of tests, illustrated in Figure 2 and performed on separate days:

1) TlimQcontrol: TlimQ measurement without a preceding cycling exercise.
2) TlimQ sham and IPS: TlimQ measurement within 2 min following the CWR test performed in a random order either with a sham ventilation or with an effective inspiratory pressure support. When necessary, CWR was arbitrary stopped at 30 min.
3) TlimQIPSiso: TlimQ measurement within 2 min following the CWR test with IPS, stopped at a time corresponding to the duration of CWR test with sham ventilation.

After completion of exercise training, the same TlimQ tests were repeated in an identical way and with the same absolute workloads. However, CWR tests were arbitrarily stopped at 30 min of cycling when the exercise endurance was dramatically increased. This condition has therefore been labeled as TlimQ@30m after exercise training.

### Training program

As a part of the ambulatory PR program, the exercise training (ET) program consisted in 16 sessions of cycling exercise with NIV using the same settings than for the evaluation tests. The sessions took place 3 times a week, and lasted 75 min per session. Upper and lower limbs muscle reinforcement was also performed during the training sessions. In addition to cycle exercise training, the program consisted in upper limbs reinforcement (bench press, arm cycle ergometer or gymnastics exercises) one session per week, and strengthening of the lower limbs (seated press or gymnastics) once a week. Finally, education sessions were proposed weekly.

Concerning the exercises on a cycle ergometer, after a 3-min warm-up period with a light load at a rate of 60 rotations per minute, the power output and the exercise duration were adjusted as follows: during the two first sessions, the patients trained at 60% of Wmax for 20 min. Exercise time was then increased to 30 min at the third session and maintained until the end of the program. Whenever a patient was able to exercise 30 min at the defined workload with a good tolerance (e.g., dyspnea below 5 on a modified Borg scale), the training intensity was increased by 5 W at the next session. This progression corresponded to an increase of 25% during the 2 first weeks, then to 11% by the end of the program.

Total training load performed per session and during the program was calculated by multiplying the workload by exercise time (watt*time in minutes).

### Statistical analysis

Data are presented as mean ± 1 standard deviation. The data were checked for normality and then compared with paired student t tests for the effect of ventilation condition or exercise training. The week to week progression of the training load was assessed by a one-way ANOVA with Bonferroni corrections. Linear regression analyses were performed between the changes in TlimQ and the training load. Considering our previous experimentation, a positive response to NIV application was set to >10% increase in TlimQ between IPS- and IPS + CWR tests, according to the reproducibility of similar endurance test reported by Ribeiro et al. (Ribeiro et al., 2015). Significance level was fixed to 5%. The statistical analysis was performed with GraphPad Prism 5 software.

Sample size calculation. In order to find out a high correlation level (r > 0.7) between the change in TlimQ with IPS and the training load, given the protective effect of IPS on TlimQ after CWR (Labeix et al., 2019), we calculated a sample size of 17 patients. To take into account drop out before the end of the rehabilitation program we decided to include 24 patients.

## Results

We analyzed the results of 20 patients who completed all the tests and the training program. The characteristics of the patients and Pulmonary Function Test results at inclusion are given in Table 1. Patients demonstrated a severe bronchial obstruction associated with pulmonary distension. Seven patients were treated with long term oxygen therapy and 1 with nocturnal NIV. Bode index was elevated at 6 ± 1, exceeding 4 in 16 patients (80%).

**TABLE 1:** Baseline characteristics and pulmonary function test results of the patients (mean ± SD).

	Measured	% Predicted
Age, years	64 ± 7	
Sex ratio (M/F)	13/7	
Height (cm)	166 ± 9	
Weight (kg)	66 ± 13	
Body mass index (kg/m^2^)	24.4 ± 6.7	
Fat free mass (kg)	49.9 ± 10.1	
FVC (l)	2.2 ± 0.8	64 ± 17
FEV1 (l)	0.75 ± 0.17	29 ± 7
FEV1/FVC	37 ± 12	
TLC (l)	7.3 ± 1.5	129 ± 21
RV (l)	5.3 ± 1.1	238 ± 53
RV/TLC (%)	68 ± 7	
MIP (cmH_2_O)	42.5 ± 15.7	48 ± 19
SNIP (cmH_2_O)	42.4 ± 19.9	47 ± 20
Resting PaO_2_ (mmHg)	62.1 ± 9.0	
Resting PaCO_2_ (mmHg)	41.8 ± 7.4	
Resting SaO_2_ (%)	91 ± 4	

### Exercise capacity and effect of exercise training

Maximal exercise capacity was severely limited with a clear ventilatory limitation to exercise (ventilatory reserve <10% in all but 1 patient) and moderate CO_2_ retention in 8 patients (Table 2). 6-MWD was moderately decreased compared to predicted value.

**TABLE 2:** Maximal functional capacity measured during incremental exercise test, walk test and quadriceps force assessment before and after pulmonary rehabilitation (PR) (mean ± SD).

	BeforePR	AfterPR	*p*-value
Maximal work rate (watt)	38 ± 21	51 ± 20	**0.0006**
Maximal work rate (% pred)	33 ± 18	44 ± 16	**0.0009**
VO_2_peak (ml/min/kg)	12.6 ± 3.1	13.3 ± 4.0	0.27
VO_2_peak (%pred)	51 ± 13	53 ± 19	0.2
VEpeak (L/min)	31.0 ± 7.1	30.6 ± 9.2	0.77
HRpeak (Beat/min)	117 ± 18	117 ± 19	0.79
HRpeak (% pred)	73 ± 12	73 ± 11	0.83
Ventilatoryreserve (%)	−9.0 ± 25.4	−5.2 ± 19.9	0.66
Δ Lactate (mmol/L)	2.1 ± 1.4	4.2 ± 5.1	0.08
Δ SpO_2_(%)	−7± 4	−8 ± 4	0.65
Δ PaO_2_(mmHg)	−7.8 ± 6.9	−9.4 ± 5.9	0.25
Δ PaCO_2_(mmHg)	4.9 ± 4.0	5.9 ± 2.5	0.39
Dyspnea Borg score	6 ± 2	7 ± 3	0.76
Fatigue Borg score	4 ± 3	4 ± 2	0.53
6-min walking distance (m)	354.4 ± 93.7	374.3 ± 93.8	0.09
6-min WD (% predicted)	69 ± 19	72 ± 19	0.3
Quadriceps 1 RM (kg)	21.5 ± 9.1	25.2 ± 10.3	**0.0001**

VO_2_peak: peak pulmonary oxygen uptake, VEpeak: peak minute ventilation, HRpeak: peak heart rate. Significant values are noted in bold.

During ET, the training load increased by 142% along the sessions, from 602 ± 264 W*min to 1,331 ± 426 W*min (*p* = 0.0001); it changes significantly every week up to session 6 (2 weeks, +84%, *p* = 0.01) then the week to week progression was no more significant.

**FIGURE 3:**
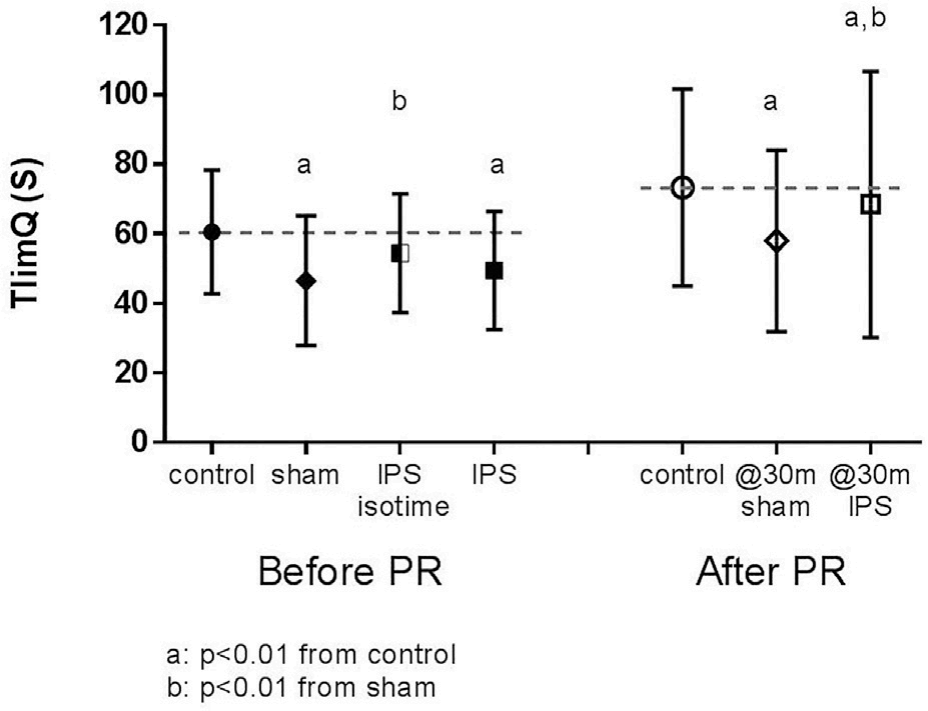
Effect of IPS and exercise training on quadriceps endurance (TlimQ). Dotted line corresponded to mean control value.

ET was associated with a significant increase in Wmax and maximal quadriceps force (Table 2). TlimQ increased with training by 13 ± 15 s (*p* = 0.0006) (Figure 3).

### Effect of IPS and training on TlimQ

Before training, IPS increased cycling endurance compared to sham CWR test (+37.5 ± 52.0%, *p* = 0.002), decreased dyspnea (-0.9 ± 2.0, *p* = 0.054) and leg fatigue (-0.9 ± 1.5, *p* = 0.02) at iso time (Table 3).

**TABLE 3:** Constant work rate exercise duration and parameters monitored at the end of the cycling test without (sham) and with effective ventilation (inspiratory pressure support, IPS), before and after training (m ± SD).

	Before training			After training at 30 m	
	Sham	IPSisotime	IPS	Sham (*n* = 18)	IPS (*n* = 18)
CWR duration (s)	767 ± 502		1011 ± 546^b^	1800^a^	1800^a^
SpO_2_(%)	91.8 ± 4.7	92.9 ± 4.3	92.6 ± 3.8	93.2 ± 4.7	94.0 ± 2.9
HR (bpm)	117 ± 20	117 ± 17	119 ± 19	116 ± 19	118 ± 18
tcPCO_2_ (mmHg)	45.1 ± 7.1	43.6 ± 7.5	43.5 ± 7.1	46.5 ± 7.6	43.5 ± 6.4
Dyspnea	5.5 ± 2.3	4.5 ± 1,9	5.5 ± 2.4	4.2 ± 2.7	4.2 ± 2.5
Fatigue	4.2 ± 1.6	3.3 ± 2,1^b^	3.9 ± 2.3	2.9 ± 2.2	3.0 ± 2.1

^a^ Different with training.

^b^ Different with effective ventilation.

The effect of IPS on TlimQ is illustrated in Figure 3. Before training, TlimQsham was significantly shortened (-14 ± 12.5s, *p* < 0.0001) compared to TlimQcontrol. TlimQIPS at isotime was not different from control condition (-6.2 ± 13.2s, *p* = 0.0501) but decreased at the end of CWR to the same extent than under sham condition. A positive effect of IPS on TlimQ at the end of CWR was noted in only 12 patients (change >10%, responder group), while in the 8 others TlimQ was not affected by IPS (non-responder group). The 2 groups did not differ significantly for their maximal exercise capacity, pulmonary function, quadriceps strength and cycling endurance.

After training, CWR test was improved similarly in sham and IPS conditions; in most of the patients (18/20) the test was arbitrarily stopped at 30 min whatever the ventilatory condition. Quadriceps endurance at 30 m in both conditions remained significantly reduced compared to TlimQcontrol, but to a larger extent in the sham condition (-9 ± 18 s for IPS vs. -21 ± 17 s, for sham, *p* = 0.004). IPS remained similarly effective to limit TlimQ variation in the responder group while it did not alter quadriceps endurance in the non-responder group.

In the whole group, we found no significant relationship between the initial change in TlimQ with IPS and the training load (*r* = -0.04, *p* = 0.9). However, in the non-responder group, we found a negative correlation between the change in TlimQ and the training load (*r* = -0.76, *p* = 0.01), suggesting that the efficacy of IPS on exercise endurance in these patients was related to the decreased respiratory work and not to an improved muscle function.

## Discussion

The main results of this study are the confirmation that NIV blunted the post-exercise decrease of quadriceps endurance in severe COPD patients, and the persistence of this benefit to a lower extent after pulmonary rehabilitation with the adjunction of a pressure ventilator support during the training sessions. However, we failed to demonstrate a relationship between the training load and the IPS-induced decrease in muscle fatigue.

### Efficacy of the exercise training program

The efficacy of the adjunct of NIV during exercise training has been emphazised in the meta-analysis by Menadue et al. (Menadue et al., 2014). In the present study, maximal exercise capacity and endurance were improved by PR confirming a training effect in this group of patients. A dramatic increase of post-training CWR, exceeding the MCID of endurance cycling test (Puente-Maestu et al., 2016), was recorded in most of the patients whatever the ventilation condition so that the tests had to be arbitrarily stopped at 30 min. We acknowledge that we did not readjust the intensity of CWR according to the improved maximal power output but the mean gain was modest (+13 W) and we do not think that it could have significantly changed the CWR duration. Maximal quadriceps force or endurance (TlimQcontrol) were also significantly improved. However, the progression of walking distance did not reach significance and an improvement above the MCID of 30 m was obtained in only 5 patients. The discordance in improvement during cycling or walking could be explained by the way the training sessions were performed (i.e., on a cycloergometer). Taken together, our results confirm a better tolerance to exercise and peripheral muscle adaptations in line with recent consensus even in severely disabled patients (McCarthy et al.).

### Effect of IPS on TlimQ

The application of NIV was associated with a significant increase in exercise tolerance, above the expected variability of a cycling endurance test (Puente-Maestu et al., 2016). Regarding the influence of NIV on quadriceps endurance, we extended our preliminary results (Labeix et al., 2019), using a more robust methodology. Indeed, the order of TlimQ measurement was randomly assigned, the patients were blinded of the experimental condition by using a sham ventilation, and the pre-training CWR test with NIV was prolonged until exhaustion. As a consequence, TlimQ IPS was significantly reduced compared to TlimQ control before training, though the benefit of NIV persisted at isotime compared to sham ventilation. Thus, when IPS alleviates the respiratory burden, peripheral muscle endurance is consistently influenced by the duration of the preceding cycling exercise. After PR, this beneficial effect of NIV on quadriceps endurance persisted but to a lower extent although the preceding CWR tests lasted the same time (30 min in almost of the patients). Then, in the trained condition, the benefit of NIV on quadriceps function is in favor of the predominant influence of the work of breathing on quadriceps fatigue (Amann et al., 2010). Our findings suggest that the interaction between ventilation and the locomotors muscles was apparent whatever the quadriceps training status. Our study was not designed to decipher this interaction in COPD patients. However, our results confirm that the measurement of quadriceps endurance after a cycling exercise represents a simple way to evidence the respiratory to peripheral muscle interaction. We acknowledge that after training, almost patients (18/20) did not reach an exercise limitation during CWR tests and that could have influenced the results of the following TlimQ, but for practical reasons it was not possible to extend cycling until the occurrence of exercise limitation (i.e. beyond 30 min).

It has also been suggested that the limitation of dynamic hyperinflation by unloading the respiratory muscles using NIV could explain the increased endurance to exercise as well as the decreased dyspnea (Gagnon et al., 2014). As a consequence, muscle O_2_ delivery increases due to the redistribution of blood flow from respiratory to limb muscles. Indeed, a lower muscle O_2_ desaturation occurred when the patients performed an incremental exercise test with a proportional assisted ventilation (Borghi-Silva et al., 2008). However, this effect on quadriceps oxygenation was not found during a constant work rate test with high pressure NIV (Gloeckl et al., 2019). Since we did not control for muscle desaturation during cycling we could not speculate on its influence on exercise-induced fatigue. Whether the reduction of dynamic hyperinflation could affect muscle function by a different way than O_2_ delivery deserves to be controlled.

Additionally, the effect of NIV on quadriceps function was noted in only 12 over 20 patients (60%). This ratio of “NIV responders” is similar to that found by our team (Labeix et al., 2019) or others on cycling endurance (van ’t Hul et al., 2004). Pulmonary function and exercise capacity were similar between responders and non-responders, and we found no factor that could predict the benefit of NIV. Moreover, ET did not change the influence of NIV to quadriceps fatigue in the non-responder group. Then, 2 patterns of response to NIV could exist in COPD patients explaining these contrasting results. Of note, such a differential response to NIV has also been demonstrated by Carrascossa et al. (Carrascossa et al., 2010) regarding ejection systolic volume during exercise.

### Relationship with training progression, utility of TlimQ assessment

Contrary to our hypothesis, we were unable to find out a relationship between the response to NIV on TlimQ and the progression of training. The day-to-day fluctuations in respiratory function and/or motivation in these severe patients explained that the goal to increase the training load could not be precisely achieved. As a consequence, we obtained an increase in the training load during the first 3 weeks in most of the patients, due to the increased in the duration of the training session. Afterwards, we adjusted weekly the training intensity but this induced a higher than expected increase in dyspnea and uncomfortable effort sensation, imposing to slow down the progression. Moreover, in some patients, we observed a dramatic decrease in training load during 1 or 2 weeks of the program. Finally, the small number of patients included as well as the heterogeneity of this group (judged on the initial exercise tolerance) could explain the absence of relationship between the training progression and the response to NIV. Such a relationship would have highlighted the utility of TlimQ assessment to select the good candidate to NIV application during PR.

While no correlation was found in the whole group, a close inverse relationship was evident in the non-responder group. Thus the decrease in work of breathing with NIV and the associated decrease in dyspnea appeared as the most important factors that influence exercise tolerance. In both groups, the effect of NIV on ET was due to the “respiratory” and non to the “locomotor muscle” positive effect.

### Limits of the study

Our study suffers from several weaknesses which could limit the extension of our findings. First, this is a small series of highly selected patients. Interferrent events during the course of the program is frequent (moderate exacerbation of COPD, variability of symptoms) which makes the patient to stop the training sessions or to reduce their intensity influencing training progression. Consequently, we performed a per-protocol analysis which restrained our conclusions. Although the training program was effective to increase some parameters of exercise tolerance, we acknowledge that the low number of exercise training sessions (16 over 6 weeks) could have restrained the benefit of PR. Twenty sessions are recommended and a longer duration of PR is advised in the more disabled or the older patients (Troosters et al., 2019). As a matter of fact, we found no significant progression of the training load after 2 weeks and a longer training program could have resulted in a more significant physical improvement.

Our study was not controlled but the cross-over design allows exploring randomly all experimental conditions.

Quadriceps endurance tests were performed in ambient air, and NIV mask was removed as soon as the patient stopped cycling. Since TlimQ involved only a limited muscle mass a cardioventilatory limitation is unlikely (Richardson, 1999), but we acknowledge that the application of NIV after an exercise bout shortens the recovery time (decrease of dyspnea) (Koopman et al., 2019) and could improve quadriceps performance. This point deserves to be confirmed.

Finally, the relative intensity imposed for the quadriceps endurance test was elevated compared to previous studies. This has probably reduced the time to exhaustion measured as a primary outcome in the current study. In their review Evans et al. (Evans et al., 2015) reported intensities ranging from 10 to 60% 1 RM to measure quadriceps endurance in COPD. We reasoned that the duration of the test should be short when breathing ambient air in order to detect an increase when NIV was applied. Moreover, since TlimQ was measured after a cycling exercise ± NIV, a lower relative intensity of the quadriceps endurance test should be associated with a longer test duration and the difficulty to discriminate between quadriceps and whole body fatigue.

## Conclusion

In severe COPD patients, quadriceps endurance is improved by an inspiratory pressure support applied during a preceding cycling endurance test and this effect remains after pulmonary rehabilitation, but to a smaller extent. Thus, the influence of unloading the respiratory muscles during exercise on trained peripheral muscles, confirms the impact of the respiratory/locomotor muscles interplay on exercise tolerance in these patients. However, this beneficial effect of NIV does not translate into a higher training load and could not help to select the good candidates for exercise training with NIV.

## Data Availability

The original contributions presented in the study are included in the article/supplementary material, further inquiries can be directed to the corresponding authors.

## Abbreviations

1RM, one maximal repetition; COPD, chronic obstructive pulmonary disease; CPET, cardio-pulmonary exercise test; CWR, constant workrate test; ET, exercise training; IPS, inspiratory pressure support; NIV, non invasive ventilation; PEEP, positive end expiratory pressure; PR, pulmonary rehabilitation; PtcO_2_, transcutaneous carbon dioxyde partial pressure; TlimQ, quadriceps endurance limit time; VO_2_, oxygen uptake; Wmax, maximal workrate.
